# A Star-PEGylation Strategy to Improve Testosterone Pharmacokinetics

**DOI:** 10.3390/molecules31010198

**Published:** 2026-01-05

**Authors:** Chae Bin Lee, Lukáš Tenora, Ruoning Zhang, Arina Ranjit, Mark C. Markowski, Barbara S. Slusher, Rana Rais

**Affiliations:** 1 Department of Neurology, Johns Hopkins University School of Medicine, Baltimore, MD 21205, USA; clee362@jh.edu (C.B.L.); aranjit4@jh.edu (A.R.); 2 Johns Hopkins Drug Discovery, Johns Hopkins University School of Medicine, Baltimore, MD 21205, USA; ltenora1@jhmi.edu (L.T.); rzhan22@jh.edu (R.Z.); 3 Johns Hopkins Sidney Kimmel Comprehensive Cancer Center, Johns Hopkins University School of Medicine, Baltimore, MD 21231, USA; 4 Departments of Psychiatry and Behavioral Sciences, Johns Hopkins University School of Medicine, Baltimore, MD 21205, USA; 5 Department of Pharmacology and Molecular Sciences, Johns Hopkins University School of Medicine, Baltimore, MD 21205, USA; 6 Department of Neuroscience, Johns Hopkins University School of Medicine, Baltimore, MD 21205, USA

**Keywords:** testosterone, star-pegylation, pharmacokinetics

## Abstract

Testosterone, an androgenic steroid hormone, regulates primary sexual characteristics and influences mood, cognition, social behavior, and sexual function. Deficiency, caused by factors such as aging and genetics, is linked to multiple disease conditions. However, current testosterone therapies are limited by extensive metabolism, poor solubility, and undesirable side effects. To address these limitations, we synthesized a four-armed star PEG-OH-linked testosterone (PEG-T). The in vitro release of testosterone from PEG-T was evaluated in buffer (pH 7.4) and mouse plasma. PEG-T was stable in the buffer, but released testosterone in plasma via esterase-mediated hydrolysis. Pharmacokinetics of testosterone and PEG-T were compared following intraperitoneal (IP) and subcutaneous (SC) administration. Following IP dosing, PEG-T exhibited a ~6-fold improvement in half-life compared to testosterone (1.18 h vs. 0.21 h), and a 54-fold increase in exposure (AUC_0-t_ = 36.0 μM·h vs. 0.67 μM·h) at equimolar doses; furthermore, following SC dosing, PEG-T showed a 4-fold improvement in both half-life (3.57 h vs. 0.91 h) and plasma exposure (11.5 μM·h vs. 3.1 μM·h). Additionally, PEG-T showed lower liver and kidney to plasma ratios, which could potentially result in reduced hepatotoxicity and nephrotoxicity. Overall, PEG-T provides sustained release pharmacokinetics, representing a promising candidate for safer testosterone replacement therapy.

## 1. Introduction

Testosterone, an androgenic steroid hormone produced by the Leydig cells in the testes [1,2], plays a crucial role in regulating primary sexual characteristics, including the maturation of sex organs and the growth of facial and body hair. In addition to its androgenic effects, testosterone also exhibits anabolic activity by promoting protein synthesis and stimulating the growth of bone and muscle [3]. Furthermore, testosterone influences mood, cognition, and social behavior [4,5,6]. Testosterone deficiency, resulting from aging, genetic factors, or diseases, is associated with various conditions, such as breast cancer, osteoporosis, Alzheimer’s disease, diabetes, and cardiovascular disorders [7,8,9,10,11].

To address these testosterone-related conditions, considerable efforts have been devoted to developing agents with enhanced anabolic activity, such as selective androgen receptor modulators (SARMs) [12]. However, no compounds have yet fully eliminated androgenic effects while preserving anabolic benefits. Consequently, testosterone remains widely used for treating conditions such as hypogonadism, male sexual impotence, and certain types of breast cancer [7,13,14]. Despite its therapeutic benefits, testosterone has notable pharmacokinetic limitations, including a short half-life, poor oral bioavailability, and extensive hepatic metabolism [15].

To overcome these challenges, various testosterone prodrugs have been developed, such as testosterone undecanoate for oral administration, and testosterone enanthate, cypionate, propionate, and undecanoate for intramuscular injections [16]. However, these formulations also have significant drawbacks. Intramuscular injections can cause supraphysiologic testosterone peaks, leading to dose-related adverse effects such as polycythemia [17,18], mood fluctuations [16], and increased sexually aggressive behavior [16]. Pellet or implant formulations are associated with extrusion, bleeding, and infection, while oral preparations frequently exhibit poor bioavailability and require multiple daily doses [15,19,20,21]. Therefore, despite the availability of several testosterone formulations and prodrug strategies, developing an ideal testosterone therapy remains an unmet medical need.

Polyethylene glycol (PEG) was first approved as a pharmaceutical excipient by the U.S. Food and Drug Administration (FDA) in the 1990s due to its demonstrated biosafety and biocompatibility [22,23,24]. PEGylation, the covalent attachment of PEG, has since been widely employed as a prodrug strategy to enhance water solubility, improve stability against enzymatic degradation, reduce uptake by the reticuloendothelial system, and facilitate targeted drug delivery to specific sites in the body [25,26,27].

Star-shaped PEG polymers (star PEGs), which consist of at least three linear arms connected to the central core, exhibit arms of equivalent length and structure. Compared to linear PEGs, star PEGs offer several advantages in drug delivery and controlled-release applications, including multiple conjugation sites and enhanced steric shielding, resulting in improved stability, prolonged circulation, and reduced premature metabolism [28,29,30] [31]. For instance, NKTR-102, a four-armed PEGylated form of CPT-11 (irinotecan), a topoisomerase Ⅰ inhibitor, demonstrated an extended half-life from 14 h to 15 days [28]. Similarly, EZN-2208, a four-armed PEGylated 7-ethyl-10-hydroxycamptothecin (SN-38), the active metabolite of CPT-11 responsible for the majority of the its cytotoxic activity, exhibited an approximately 1000-fold increase in solubility [30]. Based on these successful strategies, we employed a four-armed star-poly(ethylene glycol) (star-PEG) conjugation strategy to overcome the stability and pharmacokinetic limitations inherent to testosterone. This work represents the first application of a star-PEG scaffold to testosterone, aimed at improving its pharmacokinetic behavior and therapeutic potential.

## 2. Results and Discussions

### 2.1. Synthesis of Four-Armed PEG Testosterone

**Scheme 1:**
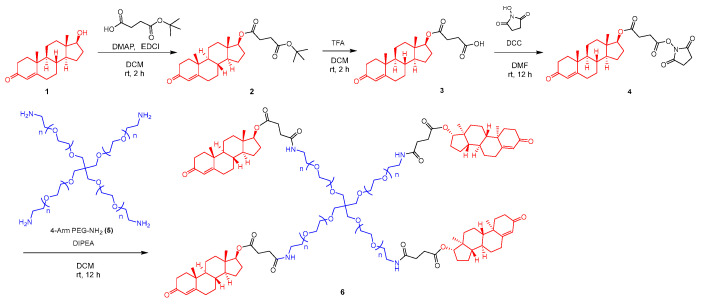
Synthesis of 4-armed PEG conjugate containing four molecules of testosterone attached via succinate handle.

Four-armed PEG testosterone, conjugate **6** (PEG-T), was synthesized in four steps (Scheme 1), with testosterone linked to PEG via a succinate spacer. Briefly, hydroxyl group in testosterone (**1**) was first esterified with partially protected *tert*-butyl succinate in presence of EDCI and DMAP to form intermediate **2**. In the next step, *tert*-butyl group was removed under acidic conditions using trifluoroacetic acid, followed by trituration with methyl *tert*-butyl ether (MTBE) to obtain compound **3**. The resulting carboxylic acid was activated as NHS ester by reaction with N-hydroxysuccinimide in the presence of DCC, leading to intermediate **4**. In the final step, four-armed PEG-NH_2_ (**5**) reacted with activated ester **4** to form target compound **6**, containing all four amino groups modified to amides with connected testosterone–succinate moieties. The desired final conjugate was isolated by preparative HPLC with a purity of 98%. The loading of testosterone in star-PEG conjugate was calculated to 7%.

### 2.2. In Vitro Release Characterization of PEG-T

**Figure 1:**
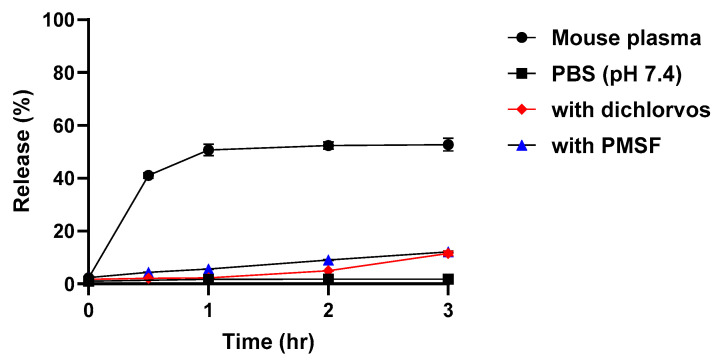
In vitro release profile of testosterone from PEG-T in mouse plasma (●), dichlorvos (♦), PMSF (▲), and PBS (pH = 7.4, ■) at 37 °C. Each datapoint represents the mean ± standard error (*n* = 3 per timepoint).

We hypothesized that pegylated testosterone (PEG-T) would exhibit slow-release profile of testosterone, as previously reported for other star-PEGs with prolonged half-life [29,30,31]. To evaluate this, we performed in vitro release studies in phosphate buffer saline (PBS; pH = 7.4) and mouse plasma under the conditions of 37 °C at a concentration equivalent to 10 μM testosterone. As shown in Figure 1, PEG-T released negligible 2% of testosterone following 3 h incubation in the PBS, but nearly 50% within 1 h in mouse plasma, suggesting esterase-mediated hydrolysis [32]. To further investigate the mechanism, mouse plasma was incubated with or without the esterase inhibitor dichlorvos and the dual esterase/serine protease inhibitor phenylmethylsulfonyl fluoride (PMSF), as rodents express multiple plasma esterases. In the presence of either inhibitor, testosterone release decreased to approximately 10%, confirming that PEG-T hydrolysis and subsequent testosterone release are primarily mediated by esterase activity.

### 2.3. Pharmacokinetic Properties of PEG-T After Intraperitoneal Administration

**Figure 2:**
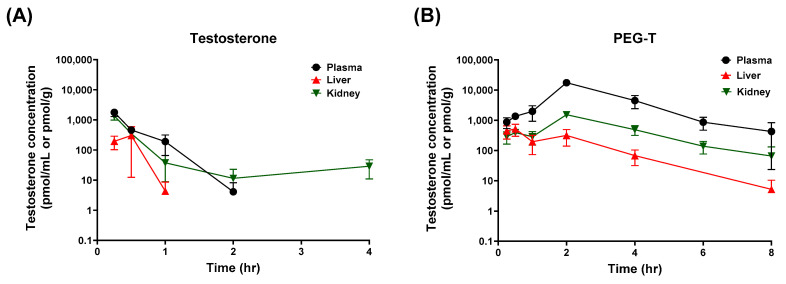
Plasma, liver, and kidney concentrations of testosterone following intraperitoneal administrations of testosterone (**A**) and PEG-T (**B**). Testosterone and PEG-T were administered at doses equivalent to 10 mg/kg of testosterone in female C57BL/6 mice. Data expressed as a mean ± standard error (SEM) (*n* = 3).

Next, we evaluated in vivo pharmacokinetics and tissue distribution (e.g., liver and kidney) of PEG-T in a head-to-head comparison with testosterone. Testosterone is an endogenous hormone with baseline levels of 11.4 ± 19.0 nM in male mice [33], which could interfere with accurate pharmacokinetic measurements. To address this, pharmacokinetic studies were conducted in female mice, where testosterone levels in serum are 20-fold lower (0.56 ± 0.56 nM) [33]. Although intravenous (IV) administration can be useful for defining intrinsic pharmacokinetic properties, and is more clinically relevant, it was not suitable for comparison in this study. Testosterone has very limited aqueous solubility compared to PEG-T, making formulation of a matched IV dose challenging, and potentially introducing vehicle-related confounders. Therefore, initial in vivo pharmacokinetic studies were conducted following intraperitoneal (IP) administration. Pharmacokinetic parameters and concentration profiles following 10 mg/kg equivalent testosterone and PEG-T intraperitoneal (IP) administration are summarized in Table 1 and Figure 2. Following IP administration, testosterone showed rapid elimination, with a half-life of 0.21 h, consistent with previously reported data [34], whereas PEG-T exhibited a 6-fold enhancement in half-life (1.18 h); furthermore, PEG-T achieved a 54-fold improvement in plasma exposures with AUC_0-t_ = 36.0 μM × h for PEG-T, compared to 0.67 μM × h for testosterone, respectively.

In addition to plasma pharmacokinetics, tissue distribution of PEG-T was evaluated in the liver and kidneys, key organs associated with drug-induced toxicity. Interestingly, PEG-T showed markedly lower tissue-to-plasma ratios compared to testosterone (0.03 vs. 0.45 for liver and 0.11 vs. 0.73 for kidney, respectively). These findings indicate that PEG-T provides sustained plasma exposure and extended half-life, while minimizing nonspecific tissue accumulation, potentially reducing the risk of hepatotoxicity and nephrotoxicity commonly associated with systemically administered testosterone [35,36].

### 2.4. Pharmacokinetic Properties of PEG-T Following Subcutaneous Administration

**Figure 3:**
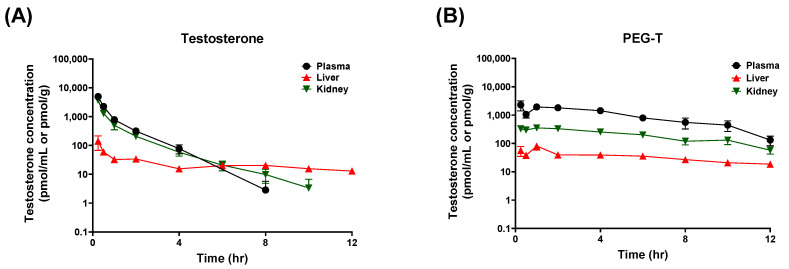
Plasma, liver, and kidney concentrations of testosterone following SC administrations of testosterone (**A**) and PEG-T (**B**). Testosterone and PEG-T were subcutaneously administered at doses equivalent to 10 mg/kg of testosterone in female C57BL/6 mice. Data expressed as a mean ± standard error (SEM) (*n* = 3).

Next, we also conducted pharmacokinetics using subcutaneous (SC) route because SC delivery is commonly used in the clinic, and known to provide sustained release of polymer–drug conjugates through depot formation at the injection site [37,38]. Studies show that SC injectable systems can significantly prolong half-life and reduce peak plasma concentrations compared with other parenteral routes [39]. Multiple testosterone formulations and routes of administration have been developed, such as intramuscular (IM) injection, transdermal gels and patches, and SC implants [40,41,42,43]. In contrast, IV administration is not clinically suitable for depot effects because it produces rapid distribution and fast clearance, with no sustained-release or depot activities. Among the available options, long-acting testosterone esters with IM injections dosed every 2 weeks remain the most popular dosing regimen, but are associated with large peak–trough fluctuations and related side effects [17,44,45]. Although ultralong-acting undecanoate ester of testosterone was developed to reduce fluctuations, its large injection volume induced a risk of pulmonary oil microembolism [46]. Furthermore, IM injections often reduce patient compliance, due to injection-related discomfort and the need for clinic visits. SC administration has gained attention due to convenience of self-administration and similar pharmacokinetic properties to IM administration [47,48,49,50]. Pharmacokinetic parameters and concentration–time profiles following SC administration of testosterone and PEG-T are summarized in Table 2 and Figure 3. Indeed, PEG-T exhibited prolonged half-life compared to unconjugated testosterone by four-fold (3.57 h vs. 0.91 h, respectively); and an additional three-fold relative to the IP route. Furthermore, PEG-T showed lower tissue/plasma ratios in liver and kidneys compared to testosterone (0.04 vs. 0.10 for liver, 0.21 vs. 0.69 for kidney, respectively), indicating reduced nonspecific tissue distribution.

Although several polymer-based delivery systems for testosterone have been investigated in preclinical settings, direct comparison with our work is limited by differences in species, study design, and dosing regimens [51,52,53]. Notably, none of these polymer-based formulations has advanced to clinical approval, underscoring ongoing translational challenges in this area.

Overall, our findings demonstrate that PEG-T maintains plasma testosterone concentrations within the physiological range for an extended duration, while reducing exposure to non-target organs. These results support subcutaneous administration as a viable and clinically relevant strategy for the further development and translation of PEG-T.

## 3. Materials and Methods

### 3.1. Materials

Mouse plasma was purchased from Innovative Research (Novi, MI, USA). Tween and dimethyl sulfoxide (DMSO) were purchased from Sigma Aldrich (St. Louis, MO, USA). All reagents used for synthesis or chromatography separation were purchased from commercial suppliers such as Sigma Aldrich. Testosterone and losartan (internal standard) were purchased from Sigma Aldrich. The solvents used were HPLC-grade and were obtained from Sigma Aldrich or Merck (Rahway, NJ, USA).

### 3.2. Synthesis and Characterization

Commercially available reagents or HPLC-grade solvents and materials were used for the synthesis of compounds described. All chemicals were reagent-grade, purchased from Sigma Aldrich (St. Louis, MO, USA), TCI, or Combi-Blocks and were used without further purification. TLC was performed on Silica gel 60 F254-coated aluminum sheets (Merck), and spots were visualized with UV light and by the solution of Ce(SO_4_)^2^.4 H_2_O (1%) and H_3_P(Mo_3_O10)^4^ (2%) in sulfuric acid (10%). Column chromatography was performed on silica gel 60 (0.040–0.063 mm, Fluka, Buchs, CH). NMR spectra were measured on Bruker AVANCE 400. 1H NMR were recorded at 401 MHz, and signals of TMS (δ 0.0, CDCl_3_), CDCl_3_ (δ 7.26) or d6-DMSO (δ 2.50, 3.33) were used for standardization. The chemical shifts are given in δ scale; the coupling constants J are given in Hz. Preparative HPLC purification was performed on an Agilent 1200 series HPLC system with an Agilent G1315D DAD detector (Agilent Technologies Inc., Santa Clara, CA, USA) Synthesis of all intermediates and final conjugates is described below. All intermediates and the final PEG-testosterone conjugate were characterized by ^1^H NMR, LC–MS, and/or gel permeation chromatography (GPC) to confirm structure, purity, and molecular weight.

#### 3.2.1. Tert-Butyl ((8R,9S,10R,13S,14S,17S)-10,13-Dimethyl-3-Oxo-2,3,6,7,8,9,10,11,12,13,14,15,16,17-Tetradecahydro-1H-Cyclopenta[a]Phenanthren-17-yl) Succinate (**2**)

Testosterone (**1**) (1.49 g, 5.17 mmol, 1 equiv.) was dissolved in anhydrous DCM (50 mL), and 4-(*tert*-butoxy)-4-oxobutanoic acid (1.00 g, 5.74 mmol, 1.1 equiv.) was added, followed by DMAP (736 mg, 6.03 mmol, 1.17 equiv.) and EDCI (1.43 g, 7.46 mmol, 1.44 equiv.). The resulting mixture was stirred at room temperature for 2 h. The reaction mixture was concentrated under reduced pressure, and the residue was purified by column chromatography on silica (petroleum ether/EtOAc; 10:1 to 5:1) to isolate compound **2** as a yellow oil (2.20 g) in 86% yield. **^1^H NMR (400 MHz, DMSO-*d_6_*):** δ 5.63 (s, 1H), 4.53 (t, *J* = 8.4 Hz, 1H), 2.48–2.43 (m, 4H), 2.42–2.33 (m, 2H), 2.28–2.21 (m, 1H), 2.19–2.11 (m, 1H), 2.08–2.01 (m, 1H), 1.96 (dd, *J* = 1.6, 13.6 Hz, 1H), 1.82–1.74 (m, 1H), 1.72–1.65 (m, 1H), 1.64–1.55 (m, 3H), 1.55–1.41 (m, 3H), 1.38 (s, 9H), 1.34–1.28 (m, 1H), 1.15 (s, 3H), 1.13–0.98 (m, 2H), 0.97–0.86 (m, 2H), 0.79 (s, 3H). **ESI MS:** 445.4 ([M + H]+).

#### 3.2.2. 4-(((8R,9S,10R,13S,14S,17S)-10,13-Dimethyl-3-Oxo-2,3,6,7,8,9,10,11,12,13,14,15,16,17-Tetradecahydro-1H-Cyclopenta[a]Phenanthren-17-yl)Oxy)-4-Oxobutanoic Acid (**3**)

To a solution of compound **2** (32.0 g, 72.0 mmol, 1 equiv.) in DCM (300 mL), TFA (164 g, 107 mL, 1.44 mol, 20 equiv.) was slowly added, and the mixture was stirred at room temperature for 1 h. Volatiles were removed in vacuo, and the residue was triturate with MTBE (20 mL), filtered, and the filter cake was concentrated to obtain compound **3** (27.0 g) as a yellow solid in 97% yield. **^1^H NMR (400 MHz, DMSO-*d_6_*):** δ 5.63 (s, 1H), 4.53 (t, *J* = 8.0 Hz, 1H), 2.47 (s, 4H), 2.38 (d, *J* = 12.4 Hz, 2H), 2.24 (d, *J* = 13.6 Hz, 1H), 2.15 (d, *J* = 16.4 Hz, 1H), 2.10–2.01 (m, 1H), 1.97–1.89 (m, 1H), 1.78 (d, *J* = 11.2 Hz, 1H), 1.69 (d, *J* = 12.0 Hz, 1H), 1.59 (s, 3H), 1.53–1.41 (m, 2H), 1.39–1.25 (m, 2H), 1.15 (s, 3H), 1.12–1.01 (m, 2H), 1.00–0.86 (m, 2H), 0.79 (s, 3H). **ESI MS:** 387.0 ([M−H]+).

#### 3.2.3. (8R,9S,10R,13S,14S,17S)-10,13-Dimethyl-3-Oxo-2,3,6,7,8,9,10,11,12,13,14,15,16,17-Tetradecahydro-1H-Cyclopenta[a]Phenanthren-17-yl (2,5-Dioxopyrroldin-1-yl) Succinate (**4**)

Compound **3** (5.00 g, 12.9 mmol, 1 equiv.) was dissolved in anhydrous DMF (50 mL), DCC (5.84 g, 5.73 mL, 28.3 mmol, 2.2 equiv.), followed by the addition of 1-hydroxypyrrolidine-2,5-dione (3.26 g, 28.3 mmol, 2.2 equiv.), and the mixture was stirred at room temperature for 12 h under nitrogen atmosphere. The precipitate (DCU) was filtered off, the filtrate was diluted with EtOAc (300 mL) and washed with dist. H_2_O (300 mL), and then the organic phase was dried over anhydrous Na_2_SO_4_ and concentrated in vacuo. The residue was purified by column chromatography on silica (petroleum ether/EtOAc; 3:7 to 1:9) to give compound **4** (4.30 g) as a colorless solid in 69% yield. **^1^H NMR (400 MHz, DMSO-*d_6_*)**: δ 5.63 (s, 1H), 4.55 (t, *J* = 8.4 Hz, 1H), 2.93 (t, *J* = 6.0 Hz, 2H), 2.80 (s, 4H), 2.66 (t, *J* = 5.6 Hz, 2H), 2.44–2.32 (m, 2H), 2.24 (d, *J* = 14.0 Hz, 1H), 2.15 (d, *J* = 16.4 Hz, 1H), 2.09–2.01 (m, 1H), 1.94 (s, 1H), 1.78 (d, *J* = 11.2 Hz, 1H), 1.69 (d, *J* = 11.6 Hz, 1H), 1.65–1.55 (m, 3H), 1.48 (s, 2H), 1.39–1.26 (m, 2H), 1.15 (s, 3H), 1.12–1.00 (m, 2H), 0.98–0.86 (m, 2H), 0.80 (s, 3H). **ESI MS:** 486.3 ([M + H]+).

#### 3.2.4. Four-Armed PEG-NH-Succinate-Testosterone Conjugate (**6**)

To a solution of 4-armed PEG-NH_2_ (3.00 g, 150 μmol, 1 equiv., Mw: 15 kDa) in anhydrous DCM (120 mL), DIPEA (38.8 mg, 52.3 μL, 300 μmol, 2 equiv.) and compound **4** (437 mg, 900 μmol, 6 equiv.) were added, and the resulting mixture was stirred at room temperature for 12 h. Volatiles were removed under vacuo, and the residue was purified by prep-HPLC (column: Welch Xltimate C4 100*30*10 um; mobile phase: [water (TFA)-MeCN]; gradient: 25–55% B over 15 min) and lyophilized to yield a colorless solid. The product was dissolved in DCM (150 mL) and basified to pH= 7 with 2M NaHCO_3_, then the organic phase was dried over anhydrous Na_2_SO_4_ and filtered. The filtrate was concentrated to remove excess solvent, then dissolved in H_2_O (20 mL) and lyophilized to give compound **6** (1.51 g) as a colorless lyophilizate in 46% yield and 98% purity. Drug loading was calculated based on molecular weights: four testosterone molecules contribute 1153.68 g/mol to the 15,000 g/mol 4-armed PEG scaffold, yielding a conjugate mass of 16,617.96 g/mol. This corresponds to a loading of 6.94%, rounded to 7%. **^1^H NMR (400 MHz, CDCl_3_):** δ 5.73 (s, 4H), 4.61 (*t*, J = 8.8 Hz, 4H), 3.65 (s, 1912 H), 2.69–2.64 (m, 8H), 2.49 (t, *J* = 6.8 Hz, 8H), 2.45–2.38 (m, 8H), 2.37–2.30 (m, 8H), 2.28–2.12 (m, 8H), 2.06–2.01 (m, 4H), 1.62–1.57 (m, 8H), 1.56–1.48 (m, 8H), 1.43–1.30 (m, 12H), 1.28–1.25 (m, 4H), 1.20 (s, 12H), 1.10–1.03 (m, 8H), 1.02 (s, 8H), 0.84 (s, 12H). **GPC:** 17,051 Mw (g/mol).

### 3.3. In Vitro Release Studies

In vitro release studies were conducted in C57BL/6 mouse plasma and phosphate-buffered saline (PBS; Ph 7.4) following previously reported methods [54]. The release of testosterone was evaluated by spiking PEG-T at a concentration equivalent to 10 μM testosterone into 1 mL of plasma or PBS. Spiked samples (in triplicates) were incubated in an orbital shaker at 37 °C for 3 h and quenched with three volumes of methanol containing internal standard (IS; losartan: 0.5 μM). Samples were vortex-mixed for 30 s and centrifuged at 10,000 *g* for 10 min at 4 °C.

Quantification of released testosterone was performed using a Dionex UPLC system coupled with Q Exactive Focus Orbitrap mass spectrometer (Thermo Fisher Scientific Inc., Waltham, MA, USA). Chromatographic separation was achieved on an Agilent Eclipse Plus column (100 mm × 2.1 mm, 1.8 μm). The autosampler temperature was maintained at 10 °C. The mobile phase consisted of acetonitrile and water, both containing 0.1% formic acid, and separation was carried out under gradient elution at a flow rate of 0.4 mL/min for 5 min. The concentration of released testosterone was determined by comparison with a 10 μM testosterone standard prepared in mouse plasma or PBS.

### 3.4. Metabolic Stability Studies Using Protease Inhibitors

Metabolic stability studies of PEG-T were conducted in mouse plasma in the presence or absence of protease-class inhibitors, as described previously [54]. Briefly, mouse plasma (1 mL) was preincubated with either phenylmethylsulfonyl fluoride (PMSF, 1.0 mM), dichlorvos (500 μM), or vehicle (control) in an orbital shaker at 37 °C for 15 min. Following preincubation, PEG-T was added to achieve a final concentration equivalent to 10 μM testosterone. Samples (*n* = 3) were incubated at 37 °C for 3 h, and aliquots were collected at predetermined time points (0, 0.5, 1, 2, 3 h). Each aliquot was quenched with three volumes of methanol containing IS (losartan, 0.5 μM), vortex-mixed for 30 s, and centrifuged at 10,000 *g* for 10 min at 4 °C. The concentration of released testosterone was quantified over time using LC-MS method, described above. Testosterone release was determined by comparison with a 10 μM testosterone standard prepared in mouse plasma.

### 3.5. Pharmacokinetic Studies in Mice

All animal studies were conducted in accordance with protocols reviewed and approved by the Institutional Animal Care and Use Committee of Johns Hopkins University (Protocol No.: M021M425). Female C57BL/6 mice (8 weeks old, 25–30 g) were maintained on a 12 h light–dark cycle, with access to food and water ad libitum. For PK studies, PEG-T and testosterone were formulated in 5% DMSO, 5% Tween 80, 90% HEPES-buffered saline (HBS; *v*/*v*/*v*) and intraperitoneally or subcutaneously administered at doses of 10 mg/kg equivalent to testosterone. All formulations were freshly prepared prior to the dosing. Mice were sacrificed at predetermined time points (0.25, 0.5, 1, 2, 4, 6 and 8 h) following drug administration. Blood samples were collected via cardiac puncture in heparinized microtubes and centrifuged at 3000× *g* for 15 min to obtain plasma. Kidney and liver tissues were excised and flash-frozen on dry ice. All collected plasma and tissue samples were stored at −80 °C until LC-MS/MS analysis.

### 3.6. Bioanalysis

Testosterone analysis was performed with developed LC-MS/MS method, modified from a previously reported method [55]. Standard and quality control (QC) stock solutions of testosterone were spiked into naïve female mouse plasma to obtain standards (0.003–30 μM) and QC (0.005–15 μM). The lower limit of quantitation (LLOQ) was 0.003 μM, and concentrations below this level were reported as below the quantification limit (BQL). Plasma samples, standards, and QCs (20 μL) were protein precipitated using methanol (100 μL) containing internal standard (0.05 μM losartan). The mixtures were vortex-mixed for 30 s and centrifuged at 10,000 *g* for 10 min at 4 °C. Following centrifugation, 100 μL of supernatant was transferred to a new microcentrifuge tube containing 50 μL of water. After vortex-mixing, the samples were centrifuged at 10,000 *g* for 10 min at 4 °C, and the supernatant was analyzed using LC-MS/MS to quantify testosterone.

For quantifying testosterone in the tissue samples, tissue samples were homogenized in methanol (5 × tissue weight) using a Geno grinder at 1500 rpm for 3 min; thereafter, 50 μL of tissue homogenates were protein precipitated using methanol (200 μL) containing IS. The subsequent sample preparation steps were identical to those described for plasma.

Quantification was performed using a Shimadzu UPLC system (Shimadzu Corporation, Kyoto, Japan) coupled with a tandem mass spectrometer QTRAP 6500 (AB SCIEX, Redwood City, CA, USA) equipped with an ESI interface operated in positive ion mode. Chromatographic separation was performed using a Trefoil CEL2 (50 × 2.1 mm, 2.6 μm, Waters, Milford, MA, USA). The mobile phase consisted of 0.1% formic acid in acetonitrile and 0.1% formic acid in water with gradient elution. Quantification was performed in multiple reaction monitoring (MRM) modes, as follows: testosterone at *m*/*z* 289.0 > 97.0 (quantitation) and 289.0 > 109.0 (confirmation); IS at *m*/*z* 423.13 > 207.12 (quantitation) and 423.13 > 377.18 (confirmation).

### 3.7. Pharmacokinetic Analysis

The plasma and tissue concentrations (nM or pmol/g) in the mice were quantified, and plots of mean plasma concentration versus time were constructed using GraphPad Prism (10.3.0, GraphPad Software, San Diego, CA, USA, 2023). Pharmacokinetic parameters were calculated by a non-compartmental analysis using Winnonlin software version 8.4 (Certara USA Inc, Princeton, NJ, USA). The peak plasma concentration (C_max_) and time to reach C_max_ (T_max_) were read directly from the experimental data. Total area under the plasma concentration-time curve from time zero to the last measured time (AUC_0-t_) was calculated using logarithmic trapezoidal rule. Tissue/plasma ratio was calculated by AUC_tissue_ divided by AUC_plasma_.

## 4. Conclusions

This study presents the first application of a multi-arm PEGylation strategy to testosterone to achieve prolonged exposure and modulation of its pharmacokinetic properties. The four-armed pegylated testosterone conjugate (PEG-T) serves as a depot-forming prodrug of testosterone, capable of releasing testosterone in vitro, through ester-mediated hydrolysis, as well as in vivo. Using two systemic routes of administration, PEG-T produced pronounced improvements in the pharmacokinetics of testosterone compared to unconjugated testosterone. Notably, PEG-T demonstrated a substantially prolonged half-life (up to 6-fold) and markedly increased systemic exposure (up to 54-fold) compared with unmodified testosterone. Moreover, PEG-T demonstrated reduced hepatic and renal distributions. These findings may help mitigate testosterone-associated hepatotoxicity and nephrotoxicity. Moreover, PEG-T maintained therapeutically relevant plasma concentrations of testosterone with minimal fluctuations, supporting its potential to lessen adverse effects linked to supraphysiological peaks and rapid clearance. Overall, PEG-T represents a promising candidate for safer and more effective testosterone replacement therapy. Future studies will focus on optimization of formulations, long-term safety, and advancement toward clinical translation.

## Figures and Tables

**Table 1:** Pharmacokinetic parameters of testosterone following IP administrations of testosterone and PEG-T (10 mg/kg equivalent). C_max_, peak concentration; AUC, area under the curve.

		C_max_ (pmol/mL or pmol/g)	Half-Life (h)	AUC_0-t_ (pmol × h/mL or pmol × h/g)	Tissue /Plasma Ratio
Testosterone	Plasma	1779.26	0.21	668.74	1
	Liver	304.87	NA	300.66	0.45
	Kidney	1364.57	0.33	487.67	0.73
PEG-T	Plasma	17,536.29	1.18	35,800.58	1
	Liver	519.05	1.21	1006.35	0.03
	Kidney	1529.98	1.38	3778.95	0.11

**Table 2:** Pharmacokinetic parameters of testosterone following SC administrations of testosterone and PEG-T (10 mg/kg equivalent). C_max_, peak concentration; AUC, area under the curve.

		C_max_ (pmol/mL or pmol/g)	Half-Life (h)	AUC_0-t_ (pmol × h/mL or pmol × h/g)	Tissue /Plasma Ratio
Testosterone	Plasma	4954.78	0.91	3025.34	1
	Liver	140.85	6.24	297.50	0.10
	Kidney	3745.38	1.49	2145.78	0.69
PEG-T	Plasma	2291.25	3.57	11,529.07	1
	Liver	81.09	5.15	411.00	0.04
	Kidney	355.42	3.79	2410.15	0.21

## Data Availability

All data are contained within the article. Further inquiries can be directed to the corresponding authors.

## Abbreviations

The following abbreviations are used in this manuscript:

^1^H NMR	Proton nuclear magnetic resonance
AUC	Area under the curve
CDCl_3_	Deuterated chloroform
CPT-11	Camptothecin-11
DCC	Dicyclohexylcarbodiimide
DCM	Dichloromethane
DCU	Dicyclohexylurea
DIPEA	N,N-diisopropylethylamine
DMAP	Dimethylaminopyridine
DMF	N,N-dimethylformamide
DMSO-*d_6_*	Deuterated dimethylsulfoxide
EDCI	1-ethyl-3-(3-dimethylaminopropyl)carbodiimide
ESI-MS	Electrospray ionization–mass spectrometry
EtOAc	Ethyl acetate
GPC	Gel permeation chromatography
HPLC	High-performance liquid chromatography
IM	Intramuscular
MeCN	Acetonitrile
MTBE	Methyl tert-butyl ether
NHS	N-hydroxysuccinimide
PEG	Polyethylene glycol
PK	Pharmacokinetics
PMSF	Phenylmethylsulfonyl fluoride
SARMs	Selective androgen receptor modulators
SC	Subcutaneous
SN-38	7-ethyl-10-hydroxycamptothecin
TFA	Trifluoroacetic acid
